# Addition of bevacizumab for malignant pleural effusion as the manifestation of acquired EGFR-TKI resistance in NSCLC patients

**DOI:** 10.18632/oncotarget.16061

**Published:** 2017-03-09

**Authors:** Tao Jiang, Aiwu Li, Chunxia Su, Xuefei Li, Chao Zhao, Shengxiang Ren, Caicun Zhou, Jun Zhang

**Affiliations:** ^1^ Department of Medical Oncology, Shanghai Pulmonary Hospital, Thoracic Cancer Institute, Tongji University School of Medicine, Shanghai, P.R. China; ^2^ Department of Lung Cancer and Immunology, Shanghai Pulmonary Hospital, Tongji University School of Medicine, Shanghai, P.R. China; ^3^ Department of Internal Medicine, Division of Hematology, Oncology and Blood & Marrow Transplantation, Holden Comprehensive Cancer Center, University of Iowa, Carver College of Medicine, Iowa, IA, USA

**Keywords:** non-small cell lung cancer, bevacizumab, EGFR TKI, EGFR mutation, T790M

## Abstract

This study aimed to investigate the role of bevacizumab in patients with advanced non-small cell lung cancer (NSCLC) who had developed acquired resistance to EGFR-TKIs therapy that manifested as malignant pleural effusion (MPE). In total, 86 patients were included. 47 patients received bevacizumab plus continued EGFR-TKIs and 39 patients received bevacizumab plus chemotherapy. The curative efficacy rate for MPE in bevacizumab plus EGFR-TKIs group was significantly higher than that in bevacizumab plus chemotherapy group (89.4% *vs*. 64.1%, respectively; *P* = 0.005). Patients in bevacizumab plus EGFR-TKIs group had longer progression-free survival (PFS) than those in bevacizumab plus chemotherapy group (median PFS 6.3 *vs*. 4.8 months, *P* = 0.042). While patients with acquired T790M mutation in bevacizumab plus EGFR-TKIs group had a significantly longer PFS than those in bevacizumab plus chemotherapy group (median PFS 6.9 *vs*. 4.6 months, *P* = 0.022), patients with negative T790M had similar PFS (median PFS 6.1 *vs*. 5.5 months, *P* = 0.588). Overall survival (OS) was similar between two groups (*P* = 0.480). In multivariate analysis, curative efficacy was an independent prognostic factor (HR 0.275, *P* = 0.047). In conclusion bevacizumab plus EGFR-TKIs could be a valuable treatment for NSCLC patients presenting with MPE upon resistant to EGFR-TKIs therapy, especially for those with acquired T790M mutation.

## INTRODUCTION

For patients with EGFR mutant non-small cell lung cancer (NSCLC), several trials have consistently demonstrated EGFR-tyrosine kinase inhibitors (TKIs) such as gefitinib, erlotinib, afatinib and icotinib can result in better outcomes than standard platinum-based chemotherapy [1-4]. Unfortunately, most patients who initially respond to EGFR-TKIs will inevitably develop resistance within 1 year [5-7]. A previous study had categorized the clinical failure modes of EGFR-TKIs into three groups namely dramatic, gradual and local progression [8]. Dramatic progression was the most common failure modes (57.3%) and most of these cases are due to the malignant pleural effusion (MPE) [8-11]. Although the recommended therapeutic strategy for NSCLC patients with dramatic progression is chemotherapy [8], NSCLC patients with MPE usually are resistant to systemic chemotherapy [12, 13].

MPE is the abnormal fluid accumulation in the pleural space, which may eventually impair the normal function of the heart and be potentially life-threatening [14, 15]. Currently, there are several management options for MPE including the tube drainage, chemical pleurodesis and use of chemotherapeutic agents. However, the relapse rate can be as high as 50%. Recently, the recombinant anti-vascular endothelial growth factor (VEGF) monoclonal antibody bevacizumab, has been shown to be efficient in suppressing the accumulation of pleural fluid [16]. Several other studies have evaluated the efficacy of bevacizumab combined with chemotherapy and the results showed that bevacizumab plus chemotherapy could achieve a higher control rate (range from 60.8% to 83.3%) of MPE than chemotherapy alone and significantly alleviate the symptom [13, 17, 18].

Besides that, bevacizumab also showed promising results in patients with EGFR sensitizing mutation. The subgroup analysis from the Chinese registration study of bevacizumab and BEYOND study showed that bevacizumab plus carboplatin and paclitaxel obtained 12.4 months PFS in patients with NSCLC and EGFR mutation, which was significantly longer than 7.9 months of the chemotherapy alone group in NSCLC patients with sensitizing EGFR mutations [19]. Several other studies also showed that bevacizumab plus EGFR-TKI had a significant longer PFS than EGFR-TKI alone for NSCLC patients with EGFR mutations with reasonable toxic-effect profiles [20-22]. The BELIEF study further suggested that bevacizumab plus EGFR-TKI seems to be preferentially effective in patients with T790M mutation in 2015 ESMO [23]. Hence, we hypothesize that bevacizumab plus EGFR-TKI might be used as a rational therapeutic option for NSCLC patients who developed acquired resistance to EGFR-TKIs that presented as MPE.

To validate our hypothesis, we retrospectively analyzed the therapeutic effect of bevacizumab in 86 Chinese EGFR mutant NSCLC patients. We compared bevacizumab plus continuation with EGFR-TKIs vs. bevacizumab plus switched chemotherapy as the subsequent treatment for MPE as the manifestation of acquisition of acquired resistance to EGFR-TKIs. In addition, their difference was further compared based on the status of acquired EGFR T790M mutation.

## RESULTS

### Patient characteristics

**Figure 1:**
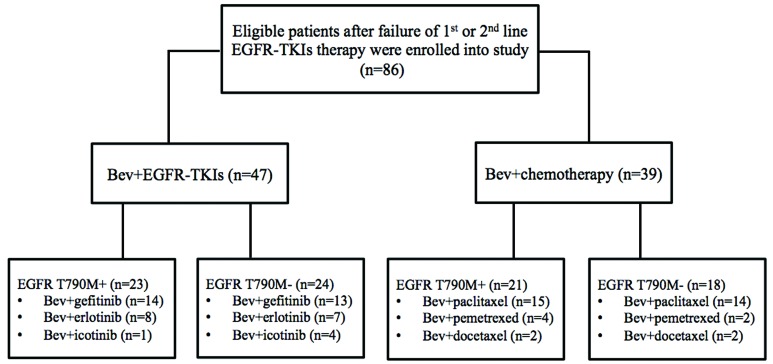
The flowchart of eligible patients enrolled into this study

A total of 86 patients who developed acquired resistance due to MPE were included in this study. 47 of them received bevacizumab plus continued EGFR-TKI and 39 received bevacizumab plus switched chemotherapy. The baseline characteristics of patients are listed in Table 1. The median patient age was 59 years (range, 45-78), and more than half of the patients were females (N = 56, 65.1%) and never-smokers (N = 63, 73.3%). All patients had histologically proven adenocarcinoma of the lung. The demographics including age, sex, smoking status, ECOG PS score, histological classification, EGFR mutation type, previous EGFR-TKIs therapy, and lines of treatment were similar between the two groups. Three EGFR-TKIs were used in the study, including gefitinib (55.8%), erlotinib (26.7%) and icotinib (17.5%). After progression on EGFR-TKIs, all of the enrolled patients with MPE received thoracentesis to drain the pleural fluid and the cells were collected by centrifugation for molecular testing (Supplemental Table S1). In total, forty-four patients showed EGFR T790M mutation. Among them, 23 patients received bevacizumab with the continuation of EGFR-TKI treatment and 21 received bevacizumab plus switched chemotherapy treatment (Figure 1).

**Table 1:** Clinical and molecular characteristics of included patients

	Bevacizumab+ EGFR-TKIs (n=47)	Bevacizumab+ chemotherapy (n=39)	Total (n=86)	*P* value
**Age (years)**				
Median	60 (45-78)	58 (52-73)	59 (45-78)	
< 65	35 (74.5%)	31 (79.5%)	66 (76.7%)	0.583
≥ 65	12 (25.5%)	8 (20.5%)	20 (23.3%)	
**Gender**				
Male	18 (38.3%)	12 (30.8%)	30 (34.9%)	0.466
Female	29 (61.7%)	27 (69.2%)	56 (65.1%)	
**Smoking history**				
Never-smoker	34 (72.3%)	29 (74.4%)	63 (73.3%)	0.833
Former/current smoker	13 (27.7%)	10 (25.6%)	23 (26.7%)	
**ECOG performance status**				
0	11 (23.4%)	16 (41.0%)	27 (31.4%)	0.080
1	29 (61.7%)	22 (56.4%)	51 (59.3%)	
2	7 (14.9%)	1 (2.6%)	8 (9.3%)	
**Histopathological classification**				
Adenocarcinoma	46 (97.9%)	38 (97.4%)	84 (97.6%)	0.559
Large-cell carcinoma	0 (0.0%)	1 (2.6%)	1 (1.2%)	
Adenosquamous carcinoma	1 (2.1%)	0 (0.0%)	1 (1.2%)	
**EGFR mutation type**				
Exon 19 deletion	26 (55.3%)	20 (51.3%)	46 (53.5%)	0.709
Exon 21 Leu858Arg mutation	21 (44.7%)	19 (48.7%)	40 (46.5%)	
**Previous EGFR-TKIs therapy**				
Gefitinib	26 (55.3%)	22 (56.4%)	48 (55.8%)	0.647
Erlotinib	12 (25.5%)	11 (28.2%)	23 (26.7%)	
Icotinib	9 (19.2%)	6 (15.4%)	15 (17.5%)	
**Treatment line of EGFR-TKIs**				
1st	32 (68.1%)	30 (76.9%)	62 (72.1%)	0.363
2nd	15 (31.9%)	9 (23.1%)	24 (27.9%)	

EGFR-TKI, epidermal growth factor receptor-tyrosine kinase inhibitor; ECOG, Eastern Cooperative Oncology Group.

### Response and survival

Response evaluation for MPE was available in all enrolled patients. In total, 78 (90.7%) patients achieved MPE control and 67 (77.9%) cases achieved complete or partial remission (CR+PR) after the addition of bevacizumab. Further analysis showed that the curative efficacy for MPE in bevacizumab plus continued EGFR-TKI group was significantly higher than that found in bevacizumab plus switched chemotherapy group (89.4% vs. 64.1%, respectively; *P* = 0.005) (Table 2).

**Table 2:** Comparison of the efficacy for malignant pleural effusion between the bevacizumab plus EGFR-TKIs and bevacizumab plus chemotherapy groups

	Bevacizumab+EGFR-TKIs (n=47)	Bevacizumab+chemotherapy (n=39)	*P* value
Complete remission	27	11	
Partial remission	15	14	
Remission not obvious	3	8	
Progressive disease	2	6	
Curative efficacy rate	42 (89.4%)	25 (64.1%)	0.005

**Figure 2:**
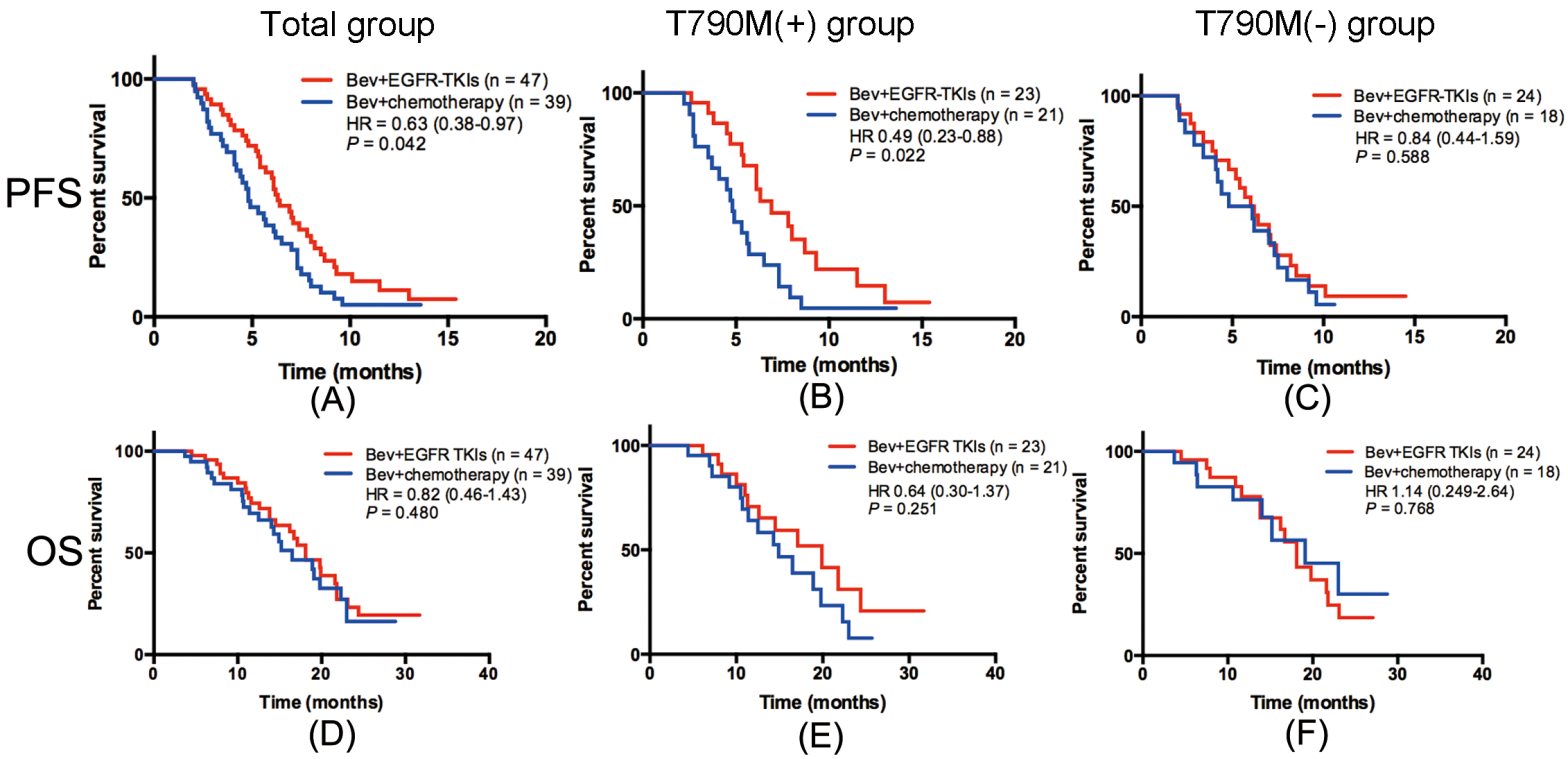
Kaplan-Meier curves for PFS and OS of included patients in different groups **A.** PFS of bevacizumab+ EGFR-TKI (B+T) vs. bevacizumab+chemotherapy (B+C) in all populations; **B.** PFS of B+T vs. B+C in patients with EGFR T790M mutation; **C.** PFS of B+T vs. B+C in patients without EGFR T790M mutation; **D.** OS of B+T vs. B+C in all populations; **E.** OS of B+T vs. B+C in patients with EGFR T790M mutation; **F.** OS of B+T vs. B+C in patients without EGFR T790M mutation.

After a median follow-up duration of 18.8 months (range, 3.5-32.7 months; last follow-up, February 2016), the median PFS for bevacizumab plus continued EGFR-TKI vs. bevacizumab plus switched chemotherapy group was 6.3 vs. 4.8 months, respectively (HR=0.63, 95% CI 0.38-0.97, *P* = 0.042) (Figure 2A). Subgroup analysis suggested that patients with EGFR T790M mutation in bevacizumab plus continued EGFR-TKI group had a significantly longer PFS than those in bevacizumab plus switched chemotherapy group (median PFS 6.9 vs. 4.6 months, HR 0.49, 95% CI 0.23-0.88; *P* = 0.022) (Figure 2B). However, patients without EGFR T790M mutation had the similar PFS between these two groups (median PFS 6.1 vs. 5.5 months, HR 0.84, 95% CI 0.44-1.59; *P* = 0.588) (Figure 2C). The median OS appeared to be longer in the bevacizumab plus continued EGFR-TKI group but this difference was not statistically significant (median OS 18.1 vs. 16.5 months, HR 0.82, 95% CI 0.46-1.43; *P* = 0.480) (Figure 2D). Patients with or without EGFR T790M mutation had the similar OS in bevacizumab plus continued EGFR-TKI vs. bevacizumab plus switched chemotherapy group (median OS 19.9 vs. 14.9 months, HR 0.64, 95% CI 0.30-1.37, *P* = 0.251; median OS 18.1 vs. 19.1 months, HR 1.14, 95% CI 0.49-2.64, *P* = 0.768; respectively).

We conducted the subgroup analyses according to types of EGFR mutation after acquired resistance. As we listed in Supplementary Table S1, there are 12 patients with EGFR exon 19 deletion plus T790M in B+T group and 10 patients with EGFR exon 19 deletion plus T790M in B+C group. Seven patients with EGFR L858R plus T790M received B+T therapy and 9 patients with EGFR L858R plus T790M received B+C therapy. Subgroup analysis showed that patients with EGFR exon 19 deletion plus T790M received B+T had significantly longer PFS than those received B+C (PFS: 7.8 vs. 4.8 month, HR=0.30, 95% CI, 0.08-0.65, P = 0.006). Patients with EGFR L858R plus T790M received B+T had longer PFS than those received B+C but it did not reach the statistical significance (PFS: 6.9 vs. 4.8 month, HR=0.37, 95% CI, 0.12-1.13, P = 0.080). OS was similar in both two groups (EGFR exon 19 deletion plus T790M group OS: 21.8 vs. 16.5 month, HR=0.80, 95% CI, 0.28-2.32, P = 0.685; EGFR L858R plus T790M group OS: 19.9 vs. 14.3 month, HR=0.40, 95% CI, 0.12-1.32, P = 0.133) (Supplemental Figure S1).

### Univariate and multivariate analyses on PFS and OS

As for PFS and OS analysis, univariate analysis showed that ECOG PS=0-1 and CR+PR correlated with longer PFS (PS=0-1: HR 0.467, 95% CI 0.201-0.911, *P* = 0.030; CR+PR: HR 0.491, 95% CI 0.250-0.852, *P* = 0.020) and OS (PS=0-1: HR 0.562, 95% CI 0.212-0.967, *P* = 0.048; CR+PR: HR 0.253, 95% CI 0.073-0.876, *P* = 0.028), and patients of never-smoking also showed a marginal longer PFS than those with smoking history (past and current) (HR 0.512, 95% CI 0.146-1.029; *P* = 0.070). In multivariate analysis, only CR+PR remained as an independent predictor of PFS (HR 0.479, 95% CI, 0.233-0.906; *P* = 0.034). The patients obtained CR+PR after treatment had a significantly lower risk of death than those obtained NC+PD (HR 0.275, 95% CI, 0.139-0.918; *P* = 0.047) (Table 3).

**Table 3:** Univariate and multivariate survival analyses in all patients

Variables	Progression-free survival				Overall survival			
	Univariate		Multivariate		Univariate		Multivariate	
	HR (95% CI)	*p* value	HR (95% CI)	*p* value	HR (95% CI)	*p* value	HR (95% CI)	*p* value
Age≤65 vs. >65	0.585 (0.214-1.242)	0.145			0.867 (0.270-2.740)	0.800		
Female vs. male	0.814 (0.435-1.481)	0.496			0.643 (0.239-1.593)	0.332		
ECOG PS 0-1 vs. 2	0.467 (0.201-0.911)	0.030	0.623 (0.283-1.518)	0.178	0.562 (0.212-0.967)	0.048	0.791 (0.260-1.510)	0.218
Never vs. ever smoker	0.512 (0.146-1.029)	0.070	0.971 (0.524-2.038)	0.221	0.236 (0.122-1.226)	0.116		
Adeno vs. non adeno	0.885 (0.332-3.367)	0.812			0.480 (0.092-1.561)	0.182		
T790M mutation vs. not	0.959 (0.514-1.788)	0.896			0.499 (0.202-1.280)	0.154		
CR+PR vs. NC+PD	0.491 (0.250-0.852)	0.020	0.479 (0.233-0.906)	0.034	0.253 (0.073-0.876)	0.028	0.275 (0.139-0.918)	0.047
1st line vs. 2nd line*	0.668 (0.267-1.430)	0.271			0.906 (0.333-2.448)	0.842		

Adeno, adenocarcinoma; Bev, bevacizumab; Chemo, chemotherapy; CR, complete remission; PR, partial remission; NC, not obvious; PD, progression disease; *, EGFR-TKIs were used as 1st or 2nd line treatment for patients with NSCLC and EGFR mutations.

### Toxicity

All of the included patients were eligible for safety analysis. The major adverse events are listed in Supplemental Table S2. Grade > 3 toxicities included rash (10.6%), paronychia (4.3%), diarrhea (6.4%), fatigue (4.3%), mucositis/stomatitis (4.3%), liver function disorder (14.9%), hypertension (19.1%), proteinuria (12.8%) in bevacizumab plus continuous EGFR-TKI group and diarrhea (5.1%), fatigue (7.7%), nausea/vomiting (2.6%), mucositis/stomatitis (2.6%), liver function disorder (5.1%), hypertension (25.6%), leukopenia (51.3%), neutropenia (53.8%), anemia (15.4%), thrombocytopenia (5.1%) in bevacizumab plus chemotherapy group.

## DISCUSSION

As far as we know, this is the first study to assess the therapeutic effect of bevacizumab in NSCLC patients who presented with MPE as the manifestation of acquired resistance to EGFR-TKI. We found that the addition of bevacizumab was effective to control MPE in NSCLC patients after failure of EGFR-TKI therapy. Moreover, we found that bevacizumab plus continued EGFR-TKI significantly improved curative efficacy of MPE and PFS, especially in patients with T790M mutations, which suggested that bevacizumab plus continued EGFR-TKI could be considered as a proper option for EGFR-TKI acquired resistance mainly presented as MPE.

MPE is one of the common progressive modes of advanced NSCLC patients with EGFR mutation receiving EGFR-TKIs and most often represents poor prognosis [16]. The current treatment options for NSCLC patients with MPE involve the tube drainage, chemical pleurodesis and intrapleural administration of chemotherapeutic agents, etc [17, 24]. However, the clinical outcome of these therapies is inconsistent. Previous study showed that VEGF is an essential mediator in the formation of pleural effusions [25], which can promote the formation of MPE by increasing the vascular permeability, stimulating the proliferation of vascular endothelial cells, promoting the efflux of plasma proteins and activating enzymes that degrade the extracellular matrix [16, 25]. Some studies have also demonstrated that bevacizumab-based chemotherapy can significantly suppress MPE than chemotherapy alone [13, 16-18]. In a prospective study, patients with NSCLC-induced MPE were randomly assigned to receive bevacizumab plus cisplatin or cisplatin alone, it was found that the curative efficacy in bevacizumab group was significantly higher than that in the cisplatin group (83.3% vs. 50.0%, *P* < 0.05) [16]. Another phase II study also confirmed that bevacizuamb plus chemotherapy had a significant effect on MPE control in NSCLC patients. The MPE control rate was 91.3% in bevacizumab with carboplatin and paclitaxel group versus 78.3% in carboplatin and paclitaxel group (*P* = 0.08) [17]. The present study was the first study to assess the efficacy of bevacizumab plus continued EGFR-TKI or switched chemotherapy in NSCLC patients who developed EGFR-TKI acquired resistance and presented as MPE. Our results have demonstrated that the addition of bevacizumab to EGFR-TKI or chemotherapy had the similar MPE control rate (90.7%). Moreover, our study further demonstrated that the addition of bevacizumab to EGFR-TKI was more effective than to chemotherapy in EGFR mutant NSCLC patients who developed EGFR-TKI acquired resistance and presented as MPE.

Theoretically, bevacizumab in combination with EGFR-TKIs might improve the anti-tumor effect because they target different tumor growth pathways (angiogenesis and EGFR activity, respectively). A previous study reported that combined blockade of the VEGF and EGFR pathways could abrogate both primary resistance to EGFR-TKIs and or acquired resistance due to T790M mutation [26]. The effect of bevacizumab plus EGFR-TKIs as first-line therapy in patients with advanced NSCLC harboring EGFR mutations has been demonstrated in the phase II trials [19-21]. In JO25567 trial, the addition of bevacizumab to erlotinib significantly prolonged PFS in NSCLC patients with EGFR mutation compared to erlotinib alone (median PFS: 16.0 vs. 9.7 months; *P* = 0.0015) [20]. The effect of bevacizumab plus EGFR-TKI was also demonstrated in the Okayama Lung Cancer Study Group Trial 1001, which suggested that bevacizumab plus gefitinib could achieve 14.4 months PFS [21]. Interestingly, our previous phase III BEYOND trial has showed that bevacizumab plus carboplatin and paclitaxel could achieve PFS of 12.4 months PFS in patient with non-squamous NSCLC and EGFR mutation [19], which was similar to the historical data of PFS using EGFR-TKIs as the first-line therapy in NSCLC patients with sensitizing EGFR mutations [2, 3]. Furthermore, the ASPIRATION study prospectively assessed continuing EGFR-TKI (erlotinib) beyond progression and showed that patients could had a median of 3.1 months PFS benefit after initial progression [27]. Taken together, these studies demonstrated that the addition of bevacizumab with continued EGFR-TKI might be used as a rational therapeutic option for NSCLC patients who developed acquired resistance to EGFR-TKIs that presented as MPE. In the current study, our results further demonstrated that bevacizumab plus EGFR-TKI or chemotherapy was also effective in NSCLC patients who presented with MPE as the manifestation of acquired resistance to EGFR-TKI. Furthermore, we have demonstrated that bevacizumab plus EGFR-TKI can have superior efficacy than bevacizumab plus chemotherapy, therefore suggested that bevacizumab plus continued EGFR-TKIs is a rational treatment for MPE upon resistance,which warrant large-scale, randomized clinical trials to validate.

Our study further performed a subgroup analysis based on the T790M mutation status. Compared to bevacizumab plus switched chemotherapy, we found that bevacizumab plus continued EGFR-TKIs significantly prolonged PFS in patients with T790M mutation but not those without this particular mutation. T790M is one of the most common mechanisms of acquired resistance to the first-generation EGFR-TKIs. To date in China, there is still no standard therapy for patients with T790M-mediated EGFR-TKI resistance although several new agents such as CO-1686 and AZD9291 that target T790M are being investigated in phase III trials (e.g. AURA3) and showed promising results in phase I/II trials [6, 28]. In a preclinical study, Naumov et al. reported that EGFR-TKI resistance could be associated with VEGF elevation in both the tumor cells and host stroma and combined blockade of the VEGF receptor and EGFR pathways could abrogate both primary resistance to EGFR-TKIs and or acquired resistance due to T790M mutation [26]. In 2015 European Cancer Congress (ECC), R.A. Stahel et al. reported that patients with EGFR T790M mutation received bevacizumab plus erlotinib had a significant longer PFS than those without EGFR T790M mutation (median PFS 16.0 vs. 10.5 months). Almost all of the subgroup analysis suggested patients with EGFR T790M mutation trended to have better PFS. Intriguingly, Furugaki K et al. reported in the xenograft models that bevacizumab plus erlotinib did not enhance antitumor activity in erlotinib primary resistant tumors with T790M mutation but did enhanced the killing when the tumors could still be suppressed by erlotinib [29]. This suggested the dependency on EGFR signaling is likely required to achieve the maximal combination effect of EGFR-TKI and bevacizumab - partly because VEGF signaling shares some of the common downstream effectors of EGFR signaling, therefore mechanisms confers primary resistance to EGFR-TKI might impair the response to VEGF inhibition too. The better combination effect observed in patients with acquired T790M mutation is probably because their cancer cells still rely heavily on EGFR and its downstream signaling for growth and survival. Further prospective trials are needed to define whether bevacizumab plus EGFR-TKIs has clinical value for patients who have primary resistance to EGFR-TKI that harbor *de novo* T790M mutation.

We must mention that we have several limitations in this study. Firstly, the sample size is small and the nature of the retrospective study may have introduced collection bias. Secondly, not all of the patients with EGFR mutations received EGFR-TKIs as first-line treatment, which will inevitably induce the imbalance in OS analysis. Thirdly, most of the patients had their EGFR T790M mutation diagnosed by using pleural effusion, which might be inconsistent with findings from the tumor tissues. Last but not least, majority of the included patients received treatment after third-line therapy but we did not collect the details of following treatment. This would result in a bias when performed the OS analysis that should be acknowledged.

In conclusion, we found that bevacizumab was effective to control MPE in patients who developed acquired resistance to EGFR-TKI. Bevacizumab plus continued EGFR-TKI resulted in better effusion control and longer PFS than bevacizumab plus switched chemotherapy, especially for patients harboring acquired EGFR T790M mutation. This observation suggested bevacizuamb plus continued EGFR-TKI should be considered as a proper regimen for patients who have failed first-line or second-line EGFR-TKIs therapy due to MPE.

## MATERIALS AND METHODS

### Study population

We retrospectively reviewed the medial records of advanced NSCLC patients with sensitizing EGFR mutation (either exon 19 deletion or Leu858Arg mutation) that had received bevacizumab therapy after the acquisition of resistance to EGFR-TKIs therapy (gefitinib, erlotinib or icotinib) from December 2011 and December 2015. Patients met the following criteria were included: 1) age > 18 years; 2) cytologically or histologically confirmed advanced NSCLC with EGFR sensitizing mutations (e.g. exon 19 deletion or L858R); 3) chest X-ray, ultrasonography or computed tomography (CT) scan showing newly developed or increased large areas of unilateral or bilateral pleural effusion or polyserositis; 4) malignant tumor cells found in the pleural fluid to confirm MPE; 5) without previous treatment of bevacizumab. All patients received gefitinib, erlotinib or icotinib orally at the recommended dose, either as the first-line therapy, or after first-line standard chemotherapy. Patients received first-line therapy got another line of chemotherapy upon resistance would be excluded. Once the patients got acquired resistance of EGFR-TKI due to MPE, their pleural fluid (50 mL) was drained for cytological evaluation and molecular mutation detection were performed once cancer cells were found in the pleural fluid. The other local therapies such as talc pleurodesis and chemotherapy were excluded into this study. Considering the situation that bevacizumab is still not covered by the health insurance system in China and AVAiL study showed that bevacizumab of 7.5 mg/kg had a similar PFS as the dose of 15 mg/kg, bevacizumab was administrated 7.5 mg/kg by intrathoracic or intravenous injection initially and then intravenously on day 1 of a 21-day cycle until progressive disease (PD) again. Major clinicopathological characteristics including demographic information, Eastern Cooperative Oncology Group performance status (ECOG PS), smoking history, clinical staging [30] and lung cancer histology (WHO classification) [31] were collected. Never smoking was defined as < 100 cigarettes in a lifetime. Smoking status, ECOG PS and age were evaluated at the time of diagnosis. The response to treatment was recorded in accordance with the Response Evaluation Criteria in Solid Tumors guidelines (version 1.1) and the survival via Kaplan-Meier method. The study was conducted with the approval of the ethics committee in Shanghai Pulmonary Hospital and a written informed consent was obtained from each participant to use his or her clinical information for research analysis. All of us confirmed that all methods were performed in accordance with the relevant guidelines and regulations.

### Treatment and response evaluation

After the development of resistance to EGFR-TKIs therapy and manifested mainly as MPE, the eligible patients received bevacizumab plus continuation of EGFR-TKIs or switched to chemotherapy and bevacizumab as the 2^nd^ or 3^rd^ line treatment. This is a retrospective study and the therapeutic regimen in this study was assigned according to the agreement between the patients’ decision and thoracic oncologists’ consultation. The chemotherapy setting was identified according to doctor’s experience and patients’ willing, economic situation and performance. The evaluation of MPE control was determined according to previous studies [13, 16, 32]. Briefly, complete remission (CR) meant the accumulated fluid had disappeared and remained stable for at least four weeks; partial remission (PR) was defined as when >50% of the accumulated fluid had disappeared, symptoms had improved, and the remaining fluid did not increase for at least four weeks; remission not obvious (NC) was considered when <50% of the accumulated fluid had disappeared; PD was considered when the accumulated fluid had increased. The curative efficacy for MPE was calculated by taking the sum of CR and PR. Baseline assessments were usually performed within 2 weeks of starting treatment after thoracentesis. A chest CT scan was performed every 2 cycles (6 weeks) in routine clinical practice or otherwise as symptoms indicated. Responses were confirmed by subsequent CT scans performed 4 to 6 weeks after the initial response documentation.

### EGFR mutation analysis

All mutational analyses were performed at the Tongji University Medical School Thoracic Cancer Institute, Shanghai. Briefly, DNA was extracted using the DNeasy Blood and Tissue Kit or the QIAamp DNA FFPE Tissue Kit (both from Qiagen, Hilden, Germany). EGFR mutations were tested by amplification refractory mutation system (ARMS) as described in our previous studies [13-35]. The kits were obtained from Amoy Diagnostics Co. Ltd., Xiamen, China.

### Statistical methods

Categorical variables were compared using chi-square tests, or Fisher’s exact tests when necessary. Student t-test was conducted for comparison of continuous variables such as the means between the two groups. OS (overall survival) was calculated from the date of lung cancer diagnosis to the date of death from any cause or was censored at the last follow-up date. PFS (progression-free survival) was defined as the time from the date of the start of treatment to the date of documented disease progression, death from any cause, or the last follow-up. Kaplan-Meier estimates were used in the analysis of the time-to-event variables, and the 95% confidence interval (CI) for the median time to event was calculated. The log-rank test was used to compare cumulative survival in the two groups. Cox proportional hazards model was used for uni- and multivariate survival analyses to calculate the hazard ratios (HR) and corresponding 95% CI. *P* values are two-sided and were considered significant when less than 0.05. All statistical analyses were performed using the SPSS statistical software, version 20.0 (SPSS Inc., Chicago, IL, USA).

## SUPPLEMENTARY MATERIALS FIGURE AND TABLES






