# Supplementary material for: Addition of bevacizumab for malignant pleural effusion as the manifestation of acquired EGFR-TKI resistance in NSCLC patients
Source: Oncotarget. 2017 Mar 9;8(37):62648–57. doi: 10.18632/oncotarget.16061 (PMC5617537; doi:10.18632/oncotarget.16061)
Supplement: Supplementary file 1 [file oncotarget-08-62648-s001.pdf]

Addition of bevacizumab for malignant pleural effusion as the manifestation of acquired EGFR-TKI resistance in NSCLC patients

Supplementary Material

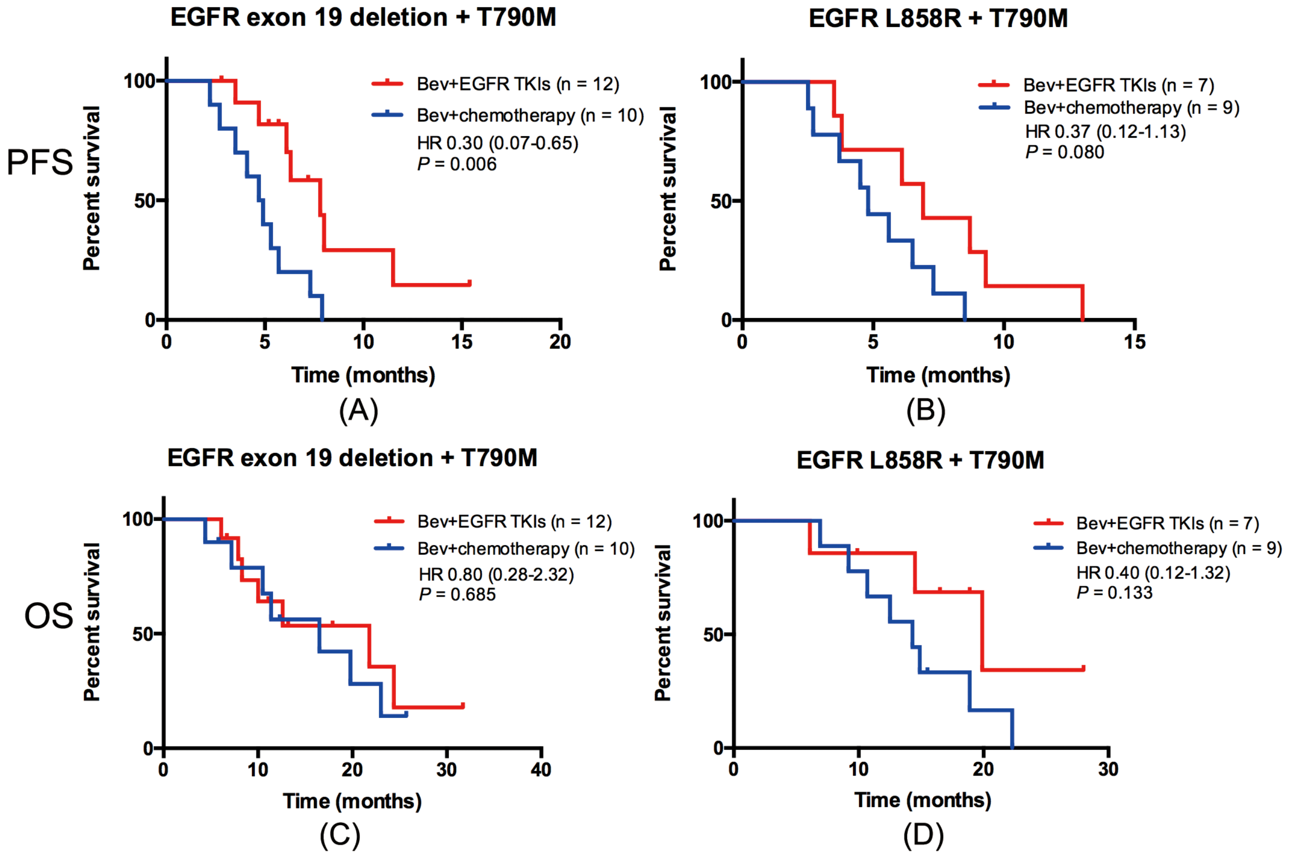

Supplemental Figure S1: Kaplan-Meier curves for PFS and OS of included patients in different groups. (A) PFS of B+T vs. B+C in patients with EGFR exon 19 deletion plus T790M; (B) PFS of B+T vs. B+C in patients with EGFR L858R plus T790M; (C) OS of B+T vs. B+C in patients with EGFR exon 19 deletion plus T790M; (D) OS of B+T vs. B+C in patients with EGFR L858R plus T790M.

For supplementary Tables 1 & 2 see in supplementary Information.
